# Adaptation of *Staphylococcus xylosus* to Nutrients and Osmotic Stress in a Salted Meat Model

**DOI:** 10.3389/fmicb.2016.00087

**Published:** 2016-02-05

**Authors:** Aurore Vermassen, Emilie Dordet-Frisoni, Anne de La Foye, Pierre Micheau, Valérie Laroute, Sabine Leroy, Régine Talon

**Affiliations:** ^1^INRA, UR454 MicrobiologieSaint-Genès Champanelle, France; ^2^INRA, INP- ENVT, Université de Toulouse, UMR 1225Toulouse, France; ^3^INRA, Plateforme d'Exploration du MétabolismeSaint-Genès Champanelle, France; ^4^Université de Toulouse, INSA, UPS, INP, LISBPToulouse, France

**Keywords:** *Staphylococcus xylosus*, meat, transcriptome, nutrient adaptation, osmotic stress

## Abstract

*Staphylococcus xylosus* is commonly used as starter culture for meat fermentation. Its technological properties are mainly characterized *in vitro*, but the molecular mechanisms for its adaptation to meat remain unknown. A global transcriptomic approach was used to determine these mechanisms. *S. xylosus* modulated the expression of about 40–50% of the total genes during its growth and survival in the meat model. The expression of many genes involved in DNA machinery and cell division, but also in cell lysis, was up-regulated. Considering that the *S. xylosus* population remained almost stable between 24 and 72 h of incubation, our results suggest a balance between cell division and cell lysis in the meat model. The expression of many genes encoding enzymes involved in glucose and lactate catabolism was up-regulated and revealed that glucose and lactate were used simultaneously. *S. xylosus* seemed to adapt to anaerobic conditions as revealed by the overexpression of two regulatory systems and several genes encoding cofactors required for respiration. In parallel, genes encoding transport of peptides and peptidases that could furnish amino acids were up-regulated and thus concomitantly a lot of genes involved in amino acid synthesis were down-regulated. Several genes involved in glutamate homeostasis were up-regulated. Finally, *S. xylosus* responded to the osmotic stress generated by salt added to the meat model by overexpressing genes involved in transport and synthesis of osmoprotectants, and Na^+^ and H^+^ extrusion.

## Introduction

Meat fermentation is an old process that relies on indigenous microbiota and is nowadays carried out under controlled conditions with addition of starter cultures. Meat starter cultures are composed of a combination of lactic acid bacteria and coagulase-negative staphylococci (Ravyts et al., 2012). *Staphylococcus xylosus* and *Staphylococcus carnosus* are the two main staphylococcal species used as starter cultures. They are associated with different species of *Lactobacillus*, such as *Lactobacillus sakei* or *Lactobacillus curvatus* or *Pediococcus*. Studies over decades of microorganisms used as starters have yielded information about their potential functionalities in meat products. Lactic acid bacteria are well-known acidifiers and bacteriocin producers that affect the technological properties and the microbial stability of products (Leroy et al., 2006). Staphylococci are recognized to play an important role in the color formation through their nitrate reductase activity (Talon and Leroy, 2006). They also contribute to the flavor development of meat products *via* their catabolism of branched-chain amino acids in odorous volatile methyl compounds (Olesen and Stahnke, 2004; Tjener et al., 2004), their antioxidant properties avoiding rancidity (Barrière et al., 2001; Rosenstein et al., 2009) and their catabolism of pyruvate in diacetyl and acetoin responsible of buttery aroma (Søndergaard and Stahnke, 2002).

The development of -omic techniques has allowed the study of the metabolism of starter cultures *in situ* in the food matrix. The transcriptomic response of *Lactococcus* in milk or cheese has been established (Cretenet et al., 2011; Taïbi et al., 2011), while the proteome of *Streptococcus thermophilus* was studied in milk and the release of bacterial proteins was studied in cheese (Gagnaire et al., 2004; Derzelle et al., 2005).

Very few studies have focused on physiology of meat starter cultures in meat matrices. They concern essentially *L. sakei*. *In vivo* expression technology (IVET) was applied to investigate gene expression of *L. sakei* during meat fermentation (Hüfner et al., 2007). This study revealed the expression of genes encoding proteins that contribute to stress-related functions. A proteomic approach highlighted that *L. sakei* overexpressed dipeptidases in the presence of sarcoplasmic extract and proteins related to energy and pyrimidine metabolism in the presence of myofibrillar extract (Fadda et al., 2010). Concerning staphylococci, the single transcriptomic concerning the response of *S. xylosus* to nitrate and nitrite in meat revealed that *S. xylosus* was subject to nitrosative stress and overcame it by expressing genes involved in iron homeostasis and antioxidant defense (Vermassen et al., 2014).

Functional genomics give us an opportunity to gain insight into the physiological and metabolic capabilities of starters *in situ* in the food matrix. As the molecular mechanisms involved in the adaptation of the bacteria in meat products are little known, we analyzed the transcriptome of *S. xylosus* in a meat model incubated for 72 h at 22°C in conditions that mimic the fermentation step. The *in situ* response of *S. xylosus* was analyzed vs. the *S. xylosus* culture used as inoculum. The results highlighted a total change in gene expression during survival of *S. xylosus* in the meat model, which reflects adaptation to nutrients and mainly to osmotic stress.

## Materials and methods

### Preparation of the inoculum and inoculation of the meat model

The *S. xylosus* C2a strain is derived from the type strain DSM20267 cured of its endogenous plasmid pSX267. Its complete genome has been sequenced (LN554884). This strain grows and behaves in meat models and in batter as strains isolated from meat products (our unpublished data). We used this laboratory strain as it is the only one of this species with some genetic background. The strain was cultured overnight at 30°C with shaking (150 rpm) in a minimal medium as already described (Fiegler and Brückner, 1997). The culture was briefly centrifuged and part of the cell pellet called “the inoculum” was immediately frozen in liquid nitrogen before extraction of RNA. Another part of the cell pellet was resuspended in physiological serum and inoculated in a pork meat model containing 0.5% glucose and 0.47 M NaCl as already described in Vermassen et al. (2014). Petri dishes were completely filled with inoculated pork meat and incubated at 22°C in a wet atmosphere for up to 72 h. These conditions probably created a gradient of oxygen in the samples. At 24, 48, and 72 h of incubation at 22°C, 200 mg meat samples were taken and immediately frozen in liquid nitrogen to stabilize the bacterial RNA. Three independent experiments were done.

To evaluate the inoculum and the growth of *S. xylosus* in meat, bacteria were enumerated after serial dilution on plates of brain-heart infusion agar, which were incubated at 30°C for 24 h.

### Physical and chemical analysis of the meat model

A digital pH meter (MP230, Mettler Toledo, Viroflay, France) with a Inlab® 413 electrode was used for measurement of meat pH.

Meat samples (2 g) were homogenized for 30 min with 8 mL of distilled water. The homogenate was then centrifuged for 20 min at 10,000 g at 4°C and the supernatant was filtered. The analyses were carried out as previously described (Nouaille et al., 2009). Briefly, glucose and fermentation products were analyzed by high-performance liquid chromatography (HPLC) using a Bio-Rad HPX87H column under the following conditions: a temperature of 48°C, 5 mM H_2_SO_4_ as the eluent at a flow rate of 0.5 mL min^−1^ and dual detection by refractometer and UV analyses. Free amino acids were analyzed after protein precipitation of the supernatant and derivatization. The derivatives were separated by HPLC on a Hypersil AA-ODS column at 40°C by a linear gradient of acetate buffer (pH 7.2) with triethylamine (0.018%), tetrahydrofurane (0.3%) and acetonitrile (60%). Diode array detectors were used at 338 and 262 nm for derivatives.

### RNA extraction, labeling and microarray analyses and validation

The RNA extraction and labeling of *S. xylosus* either from the inoculum or directly in meat after 24, 48, or 72 h of incubation were carried out as described in Vermassen et al. (2014).

A complete description of the array developed for *S. xylosus* C2a is available at the NCBI Gene Expression Omnibus (GEO) database under platform accession number GPL19201.

Microarrays were analyzed as described in Vermassen et al. (2014). Significant differences in the probe set intensities between the two conditions were identified using a linear model with an empirical Bayes method using all information probes to moderate the standard errors of the estimated log-fold changes (Smyth, 2004). The probabilities were corrected by the Benjamini-Hochberg procedure in order to control the false-discovery rate (FDR) with a *p*-value cut-off of 0.05. All the probes with an FDR = 0.05 are considered to be differentially expressed. Finally, a gene was considered to be differentially expressed if at least 50% of the corresponding probes were differentially expressed and if the ratio of expression was above 2 or lower than 0.5.

Microarray data were validated as described in Vermassen et al. (2014). The targeted genes for qPCR and primer sequences are listed in Supplementary Table 1. The analyses were performed on the same samples of RNA as used for the microarray experiments. The relative fold change of gene expression, using measured *tuf* housekeeping gene expression, was determined by the 2^−ΔΔCt^ method (Livak and Schmittgen, 2001).

### Microarray data accession number

The microarray samples and data have been deposited in the GEO database under accession number GSE69743.

## Results and discussion

### Growth and survival of *S. xylosus* in the meat model and transcriptome profile

*S. xylosus* was collected at the early stationary phase of growth in a minimal medium and was inoculated in the meat model at 7.7 log CFU/g. The growth of *S. xylosus* in the meat model was exponential until 24 h and reached 9.0 log CFU/g and remained almost at this population level until the end of the experiment (72 h).

The *in situ S. xylosus* response revealed a global change in gene expression during its survival in the meat model by comparison with the inoculum. There were 1337 (658 down- and 679 up-regulated), 1326 (659 down- and 667 up-regulated), and 1070 (540 down- and 530 up-regulated) genes differentially expressed at 24, 48, and 72 h, respectively (Supplementary Table 2). Notably, 838 genes were differentially expressed at the three times of incubation. This indicated that major transcriptional changes had occurred at 24 h and lasted throughout the incubation (72 h). These genes were classified into different functional categories: the most represented being information storage and processing, cellular processes and metabolism.

To validate the microarray analysis independently, the relative expression of 45 differentially expressed genes representing more than 5% of the common genes with significantly modified expression was measured by qPCR (Supplementary Table 1). The microarray and qPCR results for the tested genes were strongly correlated for the three times of incubation (24 h: *r*^2^ = 0.931 and slope = 1.235x, 48 h *r*^2^ = 0.918 and slope = 1.273x, 72 h *r*^2^ = 0.893 and slope = 1.394x) and the expected trend in the expression pattern was confirmed (Supplementary Figure 1).

The inoculum of *S. xylosus* was grown in a minimal medium with glucose as sole carbon source, NH_4_ as nitrogen source, 3 vitamins and trace elements. It then has to adapt its physiology to a pork meat model medium with an approximate composition (expressed as g/per 100 g) of proteins (15–22), lipids (1.5–4.0), minerals and trace amounts of carbohydrate (Toldrá, 2007) with glucose and NaCl added in our conditions. Furthermore, it has to adapt to different environmental conditions: pH variations (pH 5.9 in meat vs. 7.0), solid medium vs. liquid one.

### Cell division and cell lysis

Even though *S. xylosus* began a plateau phase at 24 h, it remained metabolically active in the meat model until 72 h, as revealed by the modulation of 60 genes involved in information processing and storage and 36 genes involved in cellular processes (cell division, cell wall/membrane biogenesis) (Table 1, Supplementary Table 2).

**Table 1:** **Genes of ***Staphylococcus xylosus*** discussed in this study differentially expressed over time in meat**.

**Gene ID**	**Gene name**	**Description**	**Mean ratio of expression**		
			**24 h**	**48 h**	**72 h**
**CELL DIVISION AND CELL LYSIS**					
**Replication, recombination, repair, and transcription**					
SXYL_01165	*dnaE*	DNA polymerase III subunit alpha	2.6	3.5	2.6
SXYL_01799		DNA polymerase X family protein	2.4	2.6	2.0
SXYL_01529-30	*parCE*	DNA topoisomerase 4 subunit A, B	5.0^*^	3.8^*^	2.9^*^
SXYL_00016	*dnaB*	Replicative DNA helicase	4.9	4.4	3.7
SXYL_01219	*ruvB*	Holliday junction ATP-dependent DNA helicase RuvB	4.2	3.4	2.5
SXYL_01659	*recG*	ATP-dependent DNA helicase RecG	3.8	4.0	2.6
SXYL_02129	*recQ1*	ATP-dependent DNA helicase RecQ	4.8	7.0	5.0
SXYL_01954	*addA*	ATP-dependent helicase/nuclease subunit A	4.0	3.4	2.4
SXYL_00005-06	*gyrAB*	DNA gyrase subunits A and B	3.7^*^	3.9^*^	2.8^*^
SXYL_02427	*mfd*	Transcription-repair-coupling factor	5.8	6.9	4.2
SXYL_01583	*mutS1*	DNA mismatch repair protein MutS	3.6	4.2	2.9
SXYL_01798	*mutS2*	Endonuclease MutS2	3.2	4.8	3.5
SXYL_02373	*fusA*	Elongation factor G	3.5	2.4	
SXYL_01273	*lepA*	Elongation factor 4	8.4	8.2	5.9
SXYL_00727	*infA*	Translation initiation factor IF-1	5.8	3.2	2.5
SXYL_01186	*infC*	Translation initiation factor IF-3	6.2	4.1	3.9
**Nucleotides transport and metabolism**					
SXYL_02522-21	*xpt, pbuX*	Xanthine phosphoribosyltransferase, Xanthine permease	7.8^*^	3.7^*^	3.6^*^
SXYL_01690	*pyrP*	Uracil permease	16.2	6.3	4.1
SXYL_02420	*hpt*	Hypoxanthine phosphoribosyltransferase	6.7	4.0	2.7
SXYL_00017	*purA*	Adenylosuccinate synthetase	13.2	8.6	5.0
SXYL_00796	*pdp*	Pyrimidine-nucleoside phosphorylase	2.0	2.8	2.5
SXYL_00821	*upp*	Uracil phosphoribosyltransferase	9.6	8.2	6.1
SXYL_01689-85	*pyrBC, carAB, pyrF*	UMP biosynthesis	7.8^*^	5.0^*^	2.9^*^
SXYL_00807	*pyrG*	CTP synthase	5.7	5.2	4.3
**Division**					
SXYL_01703	*ftsA*	Cell division protein FtsA	2.9	2.8	2.6
SXYL_01801	*zapA*	Cell division protein ZapA	2.7	2.2	2.2
SXYL_01704	*divIB*	Cell division protein DivIB	4.8	3.5	2.7
SXYL_00848		Cell division protein, FtsW/RodA/SpoVE family	4.1	2.6	2.0
SXYL_01212	*mreD*	Cell shape-determining protein MreD	3.2	3.0	2.4
SXYL_01651	*smc*	Chromosome partition protein Smc	3.5	3.6	2.2
SXYL_01699	*sepF*	Cell division protein sepF	0.3	0.2	0.3
SXYL_01710	*mraZ*	Protein MraZ	0.3	0.1	0.2
SXYL_00116	*sceD2*	Probable transglycosylase SceD 2	34.5	31.9	49.3
**Peptidoglycan synthesis**					
SXYL_00822	*mnaA*	UDP-N-acetylglucosamine 2-epimerase	8.5	6.6	4.5
SXYL_01425	*murG*	UDP-N-acetylglucosamine-tr ansferase	2.4	2.9	2.3
SXYL_02467	*sle1*	N-acetylmuramoyl-L-alanine amidase Sle1	5.7	5.2	6.1
SXYL_01706-05	*mraY, murD*	Phospho-N-acetylmuramoyl-pentapeptide-transferase, UDP-N-acetylmuramoylalanine–D-glutamate ligase	8.3^*^	5.0^*^	3.1^*^
SXYL_01914	*murE*	UDP-N-acetylmuramoyl-L-alanyl-D-glutamate–L-lysine ligase	6.2	5.2	3.6
SXYL_02166	*uppP*	Undecaprenyl-diphosphatase	2.5	2.3	2.2
SXYL_01624	*uppS*	Isoprenyl transferase	6.1	3.0	2.5
SXYL_01707	*pbp1*	Penicillin-binding protein 1	4.3	2.7	2.0
SXYL_01304	*pbp3*	Penicillin-binding protein 3	2.6	3.3	2.7
**Teichoic acid synthesis**					
SXYL_02208	*tagD*	Glycerol-3-phosphate cytidylyltransferase	0.2	0.2	0.3
SXYL_01022		Teichoic acid translocation permease protein	6.4	5.9	4.6
SXYL_01987-90	*dltDCBA*	Teichoic acid biosynthesis	7.6^*^	4.7^*^	2.6^*^
**Membrane proteins**					
SXYL_01650	*ftsY*	Signal recognition particle receptor FtsY	4.2	4.8	3.0
SXYL_00842	*yidC*	Membrane protein insertase YidC	2.7	2.0	2.1
SXYL_02419	*ftsH*	ATP-dependent zinc metalloprotease FtsH	11.7	12.0	8.2
**Lipid/Phospholipid synthesis**					
SXYL_01167-68	*accDA*	Acetyl-coenzyme A carboxylase carboxyl transferase subunit beta and alpha	3.6^*^	3.7^*^	3.0^*^
SXYL_01326-27	*accBC*	Acetyl-CoA carboxylase, biotin carboxyl carrier protein and carboxylase subunit	5.6^*^	3.2^*^	2.4^*^
SXYL_01655-54	*fabDG*	Malonyl CoA-acyl carrier protein transacylase, 3-oxoacyl-[acyl-carrier protein] reductase	8.9^*^	8.6^*^	5.1^*^
SXYL_01944-43	*fabHF*	3-oxoacyl-[acyl-carrier-protein] synthase 3 and 2	2.7^*^	2.6^*^	2.1
SXYL_01921	*fabI*	Enoyl-[acyl-carrier-protein] reductase [NADPH]	2.5	2.3	
SXYL_01656	*plsX*	Phosphate acyltransferase	13.3	9.8	6.6
SXYL_01657	*fapR*	Transcription factor FapR	8.0	5.1	3.2
**Regulator/Sensor**					
SXYL_01427-28	*arlSR*	Signal transduction histidine-protein kinase ArlS, Regulator ArlR	2.6^*^	3.9^*^	2.8^*^
SXYL_00951	*vraS*	Sensor protein VraS	3.8	4.6	2.7
**Lysis**					
SXYL_00365-67	*cidABC*	Holin-like proteins CidA and CidB, Pyruvate oxidase	23.4^*^	10.4^*^	9.1^*^
SXYL_00494	*lrgA*	Antiholin-like protein LrgA		2.6	
SXYL_01730-82		Phage proteins		13,9^*^	14.1^*^
SXYL_01785		Phage repressor-like protein	0.3	0.4	0.5
SXYL_01783		Phage antirepressor protein	2.8	24.6	22.2
**CARBOHYDRATE METABOLISM**					
SXYL_00699	*glcU*	Glucose uptake protein	10.2	7.8	5.4
**Pentose phosphate pathway**					
SXYL_00698	*gdh*	Glucose 1-dehydrogenase	3.0	3.2	2.8
SXYL_00361		Gluconolactonase	3.9	3.4	2.5
SXYL_00438-40	*gntRKP*	Gluconate Repressor, Kinase, Permease	19.8^*^	38.6^*^	16.1^*^
SXYL_00568	*rpi*	Ribose-5-phosphate isomerase		2.5	3.0
**EMBDEN-meyerhof-parnas pathway**					
SXYL_01308	*glkA*	Glucokinase	2.8	2.9	
SXYL_02069-67	*pgk, tpiA, gpmI*	Phosphoglycerate kinase, Triosephosphate isomerase, 2,3-bisphosphoglycerate-independent phosphoglycerate mutase	6.5^*^	4.5^*^	3.1^*^
**Pyruvate metabolism**					
SXYL_00250		L-lactate permease		2.5	2.6
SXYL_00170	*lqo*	L-lactate-quinone oxidoreductase	2.5		
SXYL_00276	*ldhB*	L-lactate dehydrogenase	0.2	0.3	0.4
SXYL_01023-24	*pflAB*	Formate acetyltransferase	2.2^*^	2.3^*^	2.1^*^
SXYL_01839-42	*pdhABCD*	Pyruvate dehydrogenase complex	3.7^*^	3.4^*^	2.9^*^
SXYL_00194	*lplA*	Lipoate-protein ligase A	6.4	11.3	6.1
SXYL_02616		Acetate-CoA ligase	3.5	4.3	3.6
**TCA CYCLE AND RESPIRATORY CHAIN**					
SXYL_01793-95	*sdhABC*	Succinate dehydrogenase	3.0^*^	4.4^*^	3.5^*^
SXYL_01636-37	*sucCD*	Succinyl-CoA synthase		2.5^*^	2.2^*^
SXYL_00579	*mqo*	Malate:quinone oxidoreductase	5.0	6.3	4.7
SXYL_00824-31	*atpBEFHAGDC*	F0F1-type ATP synthase	8.7^*^	5.5^*^	4.0^*^
SXYL_00823	*atpI*	Putative ATP synthase protein I	7.3	3.7	2.6
SXYL_00539-42	*narGHIJ*	Respiratory nitrate reductase	4.2^*^	4.4^*^	3.1^*^
SXYL_00534-36	*nirBD, sirB*	Nitrite reductase	6.3^*^	3.6^*^	3.3^*^
SXYL_00547	*narT*	Nitrate transporter NarT	2.3		
SXYL_00530		Formate/nitrite transporter	2.9	2.2	2.4
**Regulators**					
SXYL_00544	*nreB*	Oxygen sensor histidine kinase NreB	2.1		
SXYL_01366	*srrB*	Sensor histidine kinase SrrB	2.7	2.1	
SXYL_00883	*rex*	Redox-sensing transcriptional repressor Rex	0.4	0.4	0.4
**COFACTOR, VITAMIN SYNTHESIS**					
**Molybdenum**					
SXYL_00675-77	*modABC*	Molybdate ABC transporter	5.5^*^	5.0^*^	3.7^*^
SXYL_00682-87	*moeA, mobAB, moaAE*	Molybdenum biosynthesis	3.0^*^	3.1^*^	2.9^*^
SXYL_00679	*moeB*	Molybdopterin biosynthesis protein MoeB	2.1	1.8	1.7
**Heme**					
SXYL_01196-99	*hemCDBL1*	Heme biosynthesis	3.3^*^	4.0^*^	3.2^*^
SXYL_01274	*hemN*	Oxygen-independent coproporphyrinogen oxidase III	6.6	5.0	3.2
SXYL_00972	*hemL2*	Glutamate-1-semialdehyde 2,1-aminomutase 2	3.1	3.3	2.5
SXYL_01040-41	hemHG	Heme biosynthesis	4.2^*^	4.9^*^	5.6^*^
SXYL_01819	*ctaB*	Protoheme IX farnesyltransferase	2.8		2.5
SXYL_00751-53	*htsABC*	heme transport system	2.9^*^	2.8^*^	2.4^*^
SXYL_00755	*isdG*	Heme-degrading monooxygenase	0.2	0.2	0.3
SXYL_00749	*sfaB*	Siderophore biosynthesis protein, IucA/IucC family	0.1	0.2	0.2
SXYL_00944	*ftnA*	Ferritin	0.2	0.3	0.4
**Menaquinone**					
SXYL_01079-80	*menEC*	Menaquinone biosynthesis	2.3	2.8^*^	2.1
SXYL_01891-92	*menHD*	Menaquinone biosynthesis	3.2^*^	5.2^*^	4.2^*^
**Riboflavin**					
SXYL_01097-100	*ribDEBAH*	Riboflavin biosynthesis protein	0.2^*^	0.2^*^	0.3^*^
**Folate**					
SXYL_02414-16	*folKBP*	Folate biosynthesis	5.1^*^	5.1^*^	3.4^*^
SXYL_01203	*folC*	Tetrahydrofolate synthase	3.6	2.2	
SXYL_01872	*folD*	Bifunctional protein FolD	7.3	4.6	3.3
SXYL_01261	*aroE*	Shikimate dehydrogenase	4.4	4.3	2.7
**PEPTIDE AND AMINO ACID METABOLISM**					
**Transport**					
SXYL_00300-02		Oligopeptide ABC transporter	2.9^*^	2.9^*^	2.3^*^
SXYL_01184	*lysP*	Lysine-specific permease	7.1	6.9	4.5
SXYL_01528		Sodium:alanine symporter	5.11		
SXYL_01919		Sodium:alanine symporter	0.45		2.78
SXYL_02518	*gltT*	Proton/sodium-glutamate symporter	2.3		
**Peptidase**					
SXYL_01247-48		U32 family peptidases	5.8^*^	4.5^*^	3.2^*^
SXYL_00957	*ampS*	Leucyl aminopeptidase	3.4	2.7	2.2
SXYL_00948	*map*	Methionine aminopeptidase	3.0	3.0	2.0
**Valine, leucine, isoleucine**					
SXYL_00867-75	*ilvA, leuDCBA, ilvCNBD1*	Valine, leucine, isoleucine biosynthesis	0.3^*^	0.1^*^	0.1^*^
SXYL_02469	*ilvD2*	Dihydroxy-acid dehydratase	0.2	0.1	0.1
SXYL_01337-40	*lpdA, bkdA1A2*	alpha-keto acid dehydrogenase complex	4.9^*^	3.7^*^	2.8^*^
**Tryptophan, phenylalanine, tyrosine**					
SXYL_01383-85	*aroABC*	Aromatic acid biosynthesis	0.3^*^	0.3^*^	0.2^*^
SXYL_02022	*aroD*	3-dehydroquinate dehydratase	0.2	0.3	0.3
SXYL_01497	*trpA*	Tryptophan synthase alpha chain	0.4	0.4	0.5
SXYL_01128		DAHP synthetase-chorismate mutase	0.1	0.0	0.1
**Histidine**					
SXYL_00460-68	*hisZGDCBHAFI*	Histidine biosynthesis	0.1^*^	0.1^*^	0.1^*^
Cysteine, methionine					
SXYL_02644	*metE*	5-methyltetrahydropteroyltriglutamate–homocysteine methyltransferase	0.1	0.1	0.2
SXYL_00372		D-cysteine desulfhydrase	0.3	0.4	0.4
**Arginine**					
SXYL_00238-41	*rocD1, argCJB*	Arginine biosynthesis	0.3^*^	0.2^*^	0.3^*^
SXYL_00252	*arcB*	Ornithine carbamoyltransferase	0.3	0.3	0.4
SXYL_01961-62	*argGH*	Arginine biosynthesis	0.3^*^	0.2^*^	0.2^*^
SXYL_01355	*proC*	Pyrroline-5-carboxylate reductase	0.3	0.3	0.3
**Aspartate**					
SXYL_01002	*panD*	Aspartate 1-decarboxylase	0.4	0.2	0.2
SXYL_01558		Aspartokinase	0.4	0.3	0.4
SXYL_01373	*asnA*	L-asparaginase	2.6	2.8	2.4
**Glutamate, glutamine**					
SXYL_01964	*gluD1*	Glutamate dehydrogenase		2.3	
SXYL_02326	*gluD2*	Glutamate dehydrogenase	3.5		
SXYL_02459-61	*gltBCD*	Glutamate synthase	2.6^*^		
SXYL_00105		Membrane protein, EutH superfamily	20.3	29.3	12.9
SXYL_00106		Short-chain dehydrogenase	41.3	29.4	13.9
SXYL_00107	*glnA2*	Glutamine synthetase	41.7	29.2	15.7
SXYL_00108		Aldehyde dehydrogenase	41.3	29.5	15.4
**RESPONSE TO OSMOTIC STRESS**					
SXYL_01536	*mscL*	Large-conductance mechanosensitive channel	0.3	0.2	0.3
**Synthesis and accumulation of osmoprotectant**					
SXYL_00488-91	*opuCABCD*	Glycine betaine/carnitine/choline ABC transporter	24.8^*^	6.1^*^	4.3^*^
SXYL_00486	*lcdH*	L-carnitine dehydrogenase	22.8	5.0	3.3
SXYL_00223-26	*cudTCA, betA*	Glycine betaine/carnitine/choline ABC transporter	7.9^*^	3.7^*^	3.4^*^
SXYL_01171	*aapA*	D-serine/D-alanine/glycine transporter	2.6	2.8	2.3
SXYL_00317		D-serine/D-alanine/glycine transporter	4.9	6.1	4.4
SXYL_02528-29	*sdaAA1AB1*	L-serine dehydratase, alpha subunit and beta subunit	3.8^*^	5.0^*^	4.0^*^
SXYL_00820	*glyA*	Serine hydroxymethyltransferase	10.7	7.4	5.4
SXYL_01084	*metK*	S-adenosylmethionine synthetase	6.8	8.3	4.8
SXYL_02526		Sodium:dicarboxylate symporter	11.2	3.7	
**Na**^+^**/H**^+^ **antiporter**					
SXYL_01970-76	*mnhA1B1C1D1E1F1G1*	Na(+)/H(+) antiporter	6.1^*^	6.3^*^	3.4^*^
SXYL_02220-26	*mnhG2F2E2D2C2B2A2*	Na(+)/H(+) antiporter	4.8^*^	4.6^*^	2.8^*^
**Regulator**					
SXYL_00859	*rsbU*	Serine phosphatase RsbU, regulator of sigma subunit	4.2	2.9	2.8
SXYL_00860	*rsbV*	Anti-sigma-B factor antagonist	0.3	0.2	0.2
SXYL_00861	*rsbW*	Serine-protein kinase RsbW	0.2	0.2	0.2
**STRESS RESPONSE AND PIGMENTATION**					
SXYL_01551	*katB*	Catalase B	0.2	0.3	0.4
SXYL_02533	*katC*	Catalase C	0.2	0.2	0.2
SXYL_01572	*bsaA*	Glutathione peroxidase	0.2	0.4	0.4
SXYL_00374		Thioredoxin	0.1	0.2	0.2
SXYL_00051-54	*crtPQMN*	pigment biosynthesis	7.8^*^	6.3^*^	3.9^*^
SXYL_00358	*mvaS*	3-hydroxy-3-methylglutaryl CoA synthase	2.2		
SXYL_00359-60	*mvaCA*	Acetyl-CoA acetyltransferase, Hydroxymethylglutaryl-CoA reductase	2.4^*^	2.2^*^	
SXYL_02258-56	*mvaK1DK2*	Mevalonate kinase, Diphosphomevalonate decarboxylase, Phosphomevalonate kinase	5.0^*^	4.4^*^	2.9^*^
SXYL_00599	*fni*	Isopentenyl-diphosphate delta-isomerase	2.2	3.2	2.5
SXYL_01332	*ispA*	Geranyltranstransferase	4.6	3.8	2.5
SXYL_00309		Universal stress protein	0.1	0.2	0.2
SXYL_01162		Universal stress protein	0.2	0.2	0.2
SXYL_00196		General stress protein	0.0	0.0	0.0

* *Means of the expression of the clustered genes differentially expressed*.

The response of the information storage group consisted of 60 genes that were mostly up-regulated, 19 of which were involved in DNA replication and recombination, such as polymerases (*dnaE*, SXYL_01799), topoisomerase IV (*parCE*), helicases (*dnaB, ruvB, recG, recQ1, addA)*, gyrases (*gyrAB*) and mismatch repair (*mfd, mutS1, mutS2*) (Table 1). Thirty genes were involved in translation, such as genes encoding ribosomal proteins, amino-acyl tRNA ligases and proteins involved in synthesis of queuosines, which are modified nucleosides present in certain tRNAs (Supplementary Table 2). Furthermore, the gene *fusA* encoding the elongation factor G and the gene *lepA*, a ribosomal elongation factor that recognizes ribosomes after a defective translocation reaction and induces a back-translocation, and two genes *infA* and *infC* encoding translation initiation factors were up-regulated (Table 1).

Fourteen genes involved in nucleotide transport and metabolism were up-regulated (Table 1). This metabolism derived from the salvage of preformed extracellular nucleobases as highlighted by *pbuX* encoding a xanthine permease and *pyrP* a uracil permease. In meat, ATP is rapidly split into ADP and subsequently into AMP and IMP, which is degraded to inosine and hypoxanthine and further oxidized to xanthine (Battle et al., 2000, 2001). Other nucleotide metabolites are present in pork meat such as uracil (Battle et al., 2000). Together with the gene *pbuX*, three other genes (*xpt, hpt, purA*) encoding functions involved in the conversion of purine bases into nucleotides (IMP, XMP, GMP) were overexpressed. Adenine and guanine compounds are interconvertible through the intermediate formation of IMP. Similarly, in addition to *pyrP*, eight genes (*pdp, upp, pyrBC, carAB, pyrF, pyrG*) encoding functions involved in the production of UMP and CTP were identified. These genes encode enzymes that belong to either purine or pyrimidine salvage and interconversion pathways as well described for *Bacillus subtilis* (Switzer et al., 2002). The ability of coagulase-negative staphylococci to metabolize adenosine and inosine in a meat simulation medium has been demonstrated and these compounds may serve as potential alternative energy sources (Janssens et al., 2014).

Eight genes involved in cell division were overexpressed (Table 1). Division requires the participation of numerous proteins that can be functionally discriminated in Z-ring organizing proteins, which are involved in peptidoglycan construction and regulatory proteins (den Blaauwen, 2013). The first step is the polymerization of the FtsZ-ring with a number of Z-ring associated proteins such as FtsA and ZapA encoded by *ftsA* and *zapA*. The genes *divIB*, SXYL-00848 encoding proteins associated with septum synthesis and *mreD* involved in cell shape were up-regulated. In *S. xylosus* the genes *ftsA* and *divIB* belonged to a cluster of genes governing cell division and peptidoglycan synthesis as already established for several bacteria including *S. aureus* (Margolin, 2000). The gene *smc*, which plays a role in chromosome partitioning was up-regulated and two genes (*sepF, mraZ*) encoding proteins involved in septation and separation of one cell into two daughter cells were down-regulated. Finally, the gene *sceD2* encoding a transglycosylase potentially able to cleave peptidoglycan and affect separation of bacterial cells was highly up-regulated.

Concomitantly, 8 genes (*mnaA, murG, sle1, mraY, murD, murE, uppP, uppS*) involved in peptidoglycan synthesis and two genes (*pbp1, pbp3*) encoding penicillin-binding proteins involved in its translocation across the membrane were up-regulated (Table 1). The *arlSR* genes, which encode the two-component regulatory system ArlSR, were up-regulated. An *arlS* mutant in *S. aureus* exhibited altered activity of peptidoglycan hydrolases, which participate in important processes occurring during cell growth and division, such as cell wall synthesis, peptidoglycan turnover and recycling (Fournier and Hooper, 2000). Also, *vraS* encoding the sensor protein of the two-component system VraSR was overexpressed in *S. xylosus*. In *S. aureus*, VraSR positively modulates the regulation of the cell wall biosynthesis pathway (Kuroda et al., 2003). The *tag*D gene encoding the synthesis of teichoic acids was down-regulated in *S. xylosus*, while the gene SXYL_01022 and the *dlt* operon encoding, respectively, a permease protein involved in their translocation across the membrane and proteins involved in their alanylation were up-regulated.

Sixteen genes encoding membranous proteins were modulated, with 12 of them up-regulated (Table 1, Supplementary Table 2). Among these, we identified *ftsY*, which encodes FtsY involved in targeting and insertion of nascent membrane proteins into the cytoplasmic membrane, and *yidC*, which encodes YidC, which functions as a membrane protein insertase independent of the Sec protein–conducting channel. YidC can also assist in the lateral integration and folding of membrane proteins that insert into the membrane via the Sec pathway (Samuelson et al., 2000). The *ftsH* gene was involved in the quality control of integral membrane proteins.

In parallel, 13 genes involved in membrane lipid synthesis were up-regulated (Table 1). The mechanism of fatty acid synthesis is conserved and proceeds in two stages: initiation and elongation (Schujman and de Mendoza, 2008). The *accDA* genes encode the two subunits of carboxyltransferase, while the *accBC* genes encode the biotin acetyl CoA carboxylase, all of which are involved in the initiation step. In *B. subtilis*, the transcription of the operon *accBC* is under growth rate control, the rate of transcription decreasing with decreased growth (Marini et al., 2001). The genes *fabDG, fabHF, fabI* are involved in the elongation step, while *plsx* is involved in phospholipid synthesis. PlsX in *B. subtilis* plays an important role in the coordination of production of fatty acids and phospholipids (Schujman et al., 2003). FapR is a global transcriptional repressor that controls the expression of the *fap* regulon including all the *fab* genes and the *plsx* one (Schujman et al., 2003). The binding of FapR to its DNA targets is specifically inhibited by malonyl-CoA, a cellular pool that provides a mechanism for sensing the status of fatty acid synthesis. In our conditions, up-regulation of *acc* genes could lead to malonyl-CoA that can control the activity of FapR encoded by *fabR* up-regulated.

Fifty-one genes involved in cell lysis were modulated (Table 1, Supplementary Table 2). The *cidABC* cluster was overexpressed. In *S. aureus*, Cid and Lrg proteins are involved in the control of cell lysis (Ranjit et al., 2011). The *S. aureus cid* operon expresses two overlapping transcripts: a full-length *cidABC* transcript that is expressed at low levels during exponential growth and a *cidBC* transcript also expressed during exponential growth but at high levels (Rice et al., 2004). In *S. xylosus*, the *cidA* gene was highly overexpressed by comparison with the *cidB* and *cidC* genes (Supplementary Table 2). In *S. aureus, cidA* encodes a holin-like protein with a positive effect on murein hydrolase activity and *lrgA* encodes an antiholin-like protein with an inhibitory effect on these enzymes (Ranjit et al., 2011). In *S. aureus*, the *cidA* mutant displayed decreased lysis during biofilm formation while the *lrgAB* mutant was shown to increase lysis (Mann et al., 2009). In our conditions, the very high expression of *cidA* compared with expression of *lrgA* led us to suppose that cell lysis occurred during the survival of *S. xylosus* in the meat model.

Furthermore, a cluster of 46 genes (SXYL_01730-88) encoding phage proteins was modulated with 44 highly up-regulated at 48 and 72 h of incubation (Table 1, Supplementary Table 2). The gene SXYL_01785 encoding a phage repressor-like protein was down-regulated, while the gene SXYL_01783 coding a phage antirepressor protein was highly up-regulated. In *Salmonella* phage P22, the antirepressor overcomes the repressor protein involved in the maintenance of lysogeny (Levine et al., 1975). Consequently, in our meat model, particularly after 48 and 72 h of incubation, 44 genes involved in phage multiplication reflecting a lytic phase were highly up-regulated. Moreover, we have shown that a lytic phage can be induced by mitomycin C in *S. xylosus* C2a (data not shown).

Taken together these results (many up-regulated genes involved in DNA machinery, cellular process, cell lysis and the *S. xylosus* population remaining stable) suggested that a balance between cell division and cell lysis could be observed in our meat model up to 72 h.

### Glucose and lactate catabolized simultaneously

The concentrations of glucose added to the meat model and of lactate originating from the meat were close at T0 and decreased similarly during incubation to undetectable levels at 72 h (Table 2). Concomitantly, an increase of acetate was noted in the meat model throughout incubation associated with a small pH decrease of 0.27 after 48 h of incubation. *S. xylosus* produced mainly acetate as end-product and simultaneously metabolized glucose and lactate in the meat model. It has been shown that *S. aureus* is able to grow using glucose and lactate simultaneously to form acetate under aerobiosis (Ferreira et al., 2013).

**Table 2:** **Glucose and lactate consumption and acetate production in a meat model over time**.

**Sampling**	**Glucose/100 g**		**Lactate/100 g**		**Acetate/100 g**		**pH**
**(h)**	**mg**	**mmole**	**mg**	**mmole**	**mg**	**mmole**	
0	633	35	763	85	0	0	5.82
24	521	29	510	57	72	12	5.65
48	248	14	256	28	182	30	5.55
72	0	0	0	0	262	44	5.68

**Figure 1:**
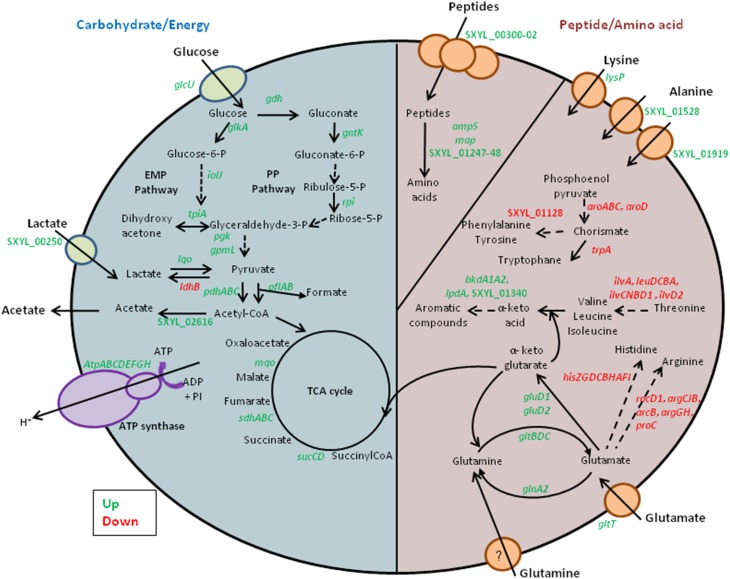
**Summary of the carbohydrate, peptide and amino acid metabolism in ***Staphylococcus xylosus*** in a meat model**.

Our transcriptomic data revealed that several genes involved in carbohydrate metabolism were overexpressed (Table 1, Figure 1). Glucose could be transported by the glucose uptake protein (GlcU). Then it could be metabolized via the glucose 1-dehydrogenase (Gdh). In *S. xylosus*, the genes *glcU* and *gdh* are co-localized and co-expressed, suggesting that GlcU recruited glucose for glucose dehydrogenase (Fiegler et al., 1999). GlcU serves in addition to PTS in taking up glucose during *S. xylosus* growth in laboratory medium (Fiegler et al., 1999). The Gdh and gluconolactonase activities resulted in formation of gluconate and consequently there was overexpression of the cluster *gntRKP*, encoding the repressor protein, gluconate kinase and gluconate permease, respectively, and involved in gluconate utilization *via* the pentose phosphate (PP) pathway (Table 1). For *Bacillus subtilis*, expression of *gnt* is derepressed in the presence of gluconate (Fujita and Fujita, 1987; Yoshida et al., 1995). In our conditions, gluconate 6-P could be further metabolized *via* the PP pathway and entered the Embden-Meyerhof-Parnas (EMP) pathway via D-glyceraldehyde 3-P (Table 1, Figure 1). In parallel, the gene *glkA* encoding glucokinase could fuel the EMP pathway with four genes up-regulated (Table 1). Deviation of part of the catabolism of glucose to the PP pathway could be an advantage as it will generate two NADH contributing to the redox status.

Finally, lactate used as carbohydrate substrate could be imported *via* the L-lactate permease encoded by the gene SXYL_00250, which is overexpressed in the meat model, and catabolized to pyruvate by the lactate-quinone oxidoreductase encoded by *lqo*, which was also overexpressed (Table 1, Figure 1). *S. aureus* consumes lactate by a lactate-quinone oxidoreductase that is critical to respiratory growth on L-lactate (Fuller et al., 2011). Interestingly, *ldhB* encoding lactate dehydrogenase was down-regulated (Table 1, Figure 1). Pyruvate could be catabolized to acetyl-CoA by both formate acetyltransferase and the pyruvate dehydrogenase complex (PDH) (Table 1). Moreover the *lplA* gene encoding a lipoate-protein ligase A responsible for lipoylation in the presence of exogenous lipoic acid, a factor required for PDH, was also up-regulated (Table 1). Acetyl CoA could therefore be catabolized to acetate by acetate-CoA ligase encoded by SXYL_02616 and excreted in the meat model as early as the first 24 h of incubation (Tables 1, 2).

In our conditions, 100% of the two carbon sources (glucose and lactate) were consumed toward EMP and PP (Table 2, Figure 1). Their catabolism generated acetyl-CoA with 55% (up to 170 mmoles) arising from lactate and 45% (up to 140 mmoles) from glucose. Acetyl-CoA can be then catabolized in acetate or feed the TCA cycle. Only small amounts of acetate were formed (Table 2). So we can hypothesize that the acetyl-CoA to acetate conversion rate did not exceed 14%.

### TCA cycle and respiratory chain

Acetyl-CoA is catabolized through the tricarboxylic acid (TCA) cycle, which is a central pathway for the catabolism of carbohydrate, fatty and amino acids, which enter the cycle at several points. Some TCA cycle genes were up-regulated in the meat model, such as the succinate dehydrogenase (*sdhABC*), succinyl-CoA synthase (*sucCD*), and malate:quinone oxidoreductase (*mqo*) genes (Table 1). For *S. aureus* in biofilm, it was found that the up-regulation of succinate dehydrogenase genes is an advantage under oxygen-limited conditions (Gaupp et al., 2010). The succinate dehydrogenase is involved in the electron transfer of the respiratory chain driving ATP synthesis. We observed that 8 genes encoding the F_0_F_1_-type ATP synthase (*atpBEFHAGDC*) and the gene *atpI*, upstream of the *atp* genes and encoding a putative ATP synthase protein I, were highly overexpressed and could furnish energy for the survival of *S. xylosus* in meat (Table 1, Figure 1).

The overexpression of several genes involved in the EMP and PP pathways, of the clusters *nar* and *nir* related to nitrate respiration, although nitrate was not present, of the nitrate and formate/nitrite transporter genes and of *pfl* encoding formate acetyl transferase, suggested anaerobic conditions (Table 1), as already mentioned for *S. aureus* grown *in vitro* in the absence of oxygen (Fuchs et al., 2007). The two-component regulatory system NreBC, which is considered as an oxygen-sensing system, stimulates the expression of genes of nitrate respiration under anaerobic conditions in *S. carnosus* and *S. aureus* (Schlag et al., 2008; Reinhart et al., 2010). The targets are the genes encoding the nitrate and nitrite reductases and the nitrate transporter. In our conditions, the regulatory gene *nreB* was up-regulated at 24 h of growth, thus explaining the up-regulation of *nar, nir*, and *narT* (Table 1). Moreover, *ssrB* encoding a sensor that autophosphorylates in the absence of O_2_ was up-regulated. This two-component system SrrAB is induced under oxygen-limiting conditions in staphylococci (Green et al., 2014). In *S. aureus*, a redox-sensing transcriptional repressor Rex is involved in the regulation of anaerobic gene expression (Pagels et al., 2010). The binding activity of Rex is enhanced by NAD^+^ while NADH decreases it. Rex regulates the expression of pathways that lead to anaerobic NAD^+^ regeneration, nitrate respiration and ATP synthesis. In our study, the transcription of *rex* was down-regulated and could explain the overexpression of genes involved in EMP and PP pathways, nitrate respiration and ATP synthesis.

### Cofactor, vitamin synthesis

In the meat model, *S. xylosus* overexpressed the cluster *modABC* encoding the molybdate ABC-type transporter and six genes, including *moeB*, of the cluster involved in the synthesis of the molybdenum cofactor (Table 1). In *S. carnosus*, anaerobic growth conditions enhanced transcription of *moeB* (Neubauer et al., 1998). Moreover, *S. carnosus* mutants defective either in *modABC* or in *moeB* were defective in nitrate reductase activity, the nitrate reductase being a molybdoenzyme (Neubauer et al., 1999). As we mentioned above, *S. xylosus* in the meat model also up-regulated the cluster *nar* encoding the nitrate reductase.

The cluster *hemCDBL1* and five other genes (*hemN, hemL2, hemHG, ctaB*) encoding proteins involved in the synthesis of heme were up-regulated (Table 1). Heme is vital to many biological systems and a multitude of redox enzymes engage heme as a catalyst of electron transfer. *S. aureus* can employ heme as a cofactor required for respiration, both by aerobic electron transport and by the anaerobic nitrate reductase complex (von Eiff et al., 2006). Moreover, the cluster *htsABC* encoding a heme transport system was up-regulated in *S. xylosus* (Table 1). In *S. aureus*, inactivation of HtsABC reduced its ability to import heme iron (Skaar et al., 2004). Heme can be an iron source, but in the meat model *S. xylosus* down-regulated the gene *isdG* encoding a heme-degrading monooxygenase that promotes catalytic degradation of heme with subsequent release of iron. Furthermore, there was down-regulation of *sfaB* belonging to the cluster *sfa* involved in synthesis of the siderophore staphyloferrin A and of *ftnA* involved in iron storage (Table 1). Thus, in the meat model, i.e., in iron-replete conditions, the synthesis of heme is more probably intended for proteins involved in respiration, such as succinate dehydrogenase encoded by *sdh* (Table 1). The genes *menEC* and *menHD* involved in synthesis of menaquinone from chorismate were overexpressed (Table 1). Menaquinone is an important cofactor that is exploited in electron transport pathways. Menaquinone biosynthesis in facultative anaerobes is increased by anaerobiosis and in *S. aureus* menaquinone deficiency is accompanied by impaired nitrate respiration (Bentley and Meganathan, 1982). During respiration, menaquinone donate electrons to the heme molecules located within cytochromes (Wakeman et al., 2012).

The cluster *ribDEBAH* involved in riboflavin synthesis was down-regulated in our conditions (Table 1). The active forms of riboflavin in the cell are flavin mononucleotide (FMN) and flavin adenine dinucleotide (FAD), but riboflavin can be found as free compounds (Perkins and Pero, 2002). In contrast, the cluster *folKBP* and 3 other genes involved in the synthesis of folate were overexpressed (Table 1). Cells require folate cofactors as acceptor/donor of one carbon unit in numerous processes such as methionine, purine, and thymine synthesis and in some degradative reactions (Rossi et al., 2011).

### Peptide and amino acid metabolism

Proteins are the main components of meat. They are classified as water-soluble (sarcoplasmic, 30%), soluble in high salt concentration (myofibrillar, 55%) and insoluble (stromal proteins, proteins from connective tissue) (Lafarga and Hayes, 2014). The sarcoplasmic and myofibrillar proteins are hydrolyzed during fermentation and ripening of fermented sausages (Hughes et al., 2002). The initial degradation is essentially due to the activity of endogenous proteinases that release peptides that can be further hydrolyzed by bacterial enzymes (Hughes et al., 2002). These peptides can be transported into *S. xylosus* by the oligopeptide transport system encoded by the three genes, SXYL_00300-302 (Table 1, Figure 1). Peptides played a key role in bacterial nutrition; their transport is generally regulated at the transcriptional level by intracellular pools of amino acids and by environmental changes such as anaerobic conditions (Hiron et al., 2007). Two genes (SXYL_01247-48) encoding peptidases from U32 family and the genes *ampS* and *map* encoding leucyl- and methionine- aminopeptidases, respectively, were overexpressed. These peptidases will furnish amino acids for *S. xylosus* (Table 1). The starter cultures (*Pediococcus pentosaceus* plus *S. xylosus* or *Lactobacillus sakei* plus *S. carnosus*) influenced the proteolytic activity and the release of amino acids in sausages (Hughes et al., 2002; Aro Aro et al., 2010). Moreover, *L. sakei* overexpressed two dipeptidases in the presence of sarcoplasmic proteins (Fadda et al., 2010).

The meat model contained variable levels of free amino acids with a broad pattern of 19 amino acids (Table 3). The total initial concentration (157 mg/100 g) increased up to 24 h and could be mainly attributed to endogenous meat peptidases. After 24 h there was a decrease of the total free amino acid content. Notably, the level of lysine decreased after 24 h and *lysP* encoding a lysine-specific permease was overexpressed in *S. xylosus* (Tables 1, 3). Likewise, the concentration of alanine decreased sharply in the meat model after 48 h of incubation and two genes encoding sodium:alanine symporters were modulated during the incubation (Tables 1, 3). In parallel, in the meat model, *S. xylosus* down-regulated 26 genes involved in the synthesis of branched-chain amino acids (nine genes belonging to the cluster *ilv-leu* and the gene *ilvD2*), aromatic amino acids (*aroABC, aroD, trpA*, SXYL_01128), histidine (*hisZGDCBHAFI*) and cysteine-methionine (*metE*, SXYL_00372) (Table 1, Figure 1). In *S. aureus*, CodY contributes to the regulation of more than 200 genes including most of the ones listed above (Majerczyk et al., 2010). CodY repression occurs in *Bacillus* cells growing in a medium containing glucose and amino acids (Shivers and Sonenshein, 2004). Furthermore, *S. xylosus* down-regulated 8 genes involved in synthesis of arginine (*rocD1*-*argCJB, arcB, argGH, proC*) (Table 1). In *S. aureus*, arginine biosynthesis is regulated through the global regulator CcpA and it was shown that a mutant *ccpA* facilitates the synthesis of arginine *via* the urea cycle (Nuxoll et al., 2012).

**Table 3:** **Concentration of free amino acids in a meat model over time**.

**Amino**	**0 h**	**24 h**	**48 h**	**72 h**
**Acid**	**mg/100 g**	**mg/100 g**	**mg/100 g**	**mg/100 g**
VAL	7.4	8.8	8.2	5.0
LEU	8.1	10.5	9.7	5.0
ILE	3.8	3.3	2.9	1.0
THR	8.0	7.9	5.7	1.4
TYR	3.8	6.1	6.2	7.3
PHE	5.0	7.6	7.9	12.4
HIS	0.0	0.0	6.9	7.6
ASP	3.6	3.7	3.1	2.9
ASN	2.9	4.3	4.3	2.1
GLU	16.0	15.7	14.8	17.5
GLN	20.5	16.9	12.0	2.1
SER	5.4	5.4	3.5	0.8
GLY	12.8	8.7	3.6	0.0
ALA	27.6	34.5	27.3	4.0
ARG	6.7	11.3	11.4	6.1
CYS	1.4	1.1	0.8	0.6
MET	3.3	4.9	5.3	6.4
LYS	16.8	19.1	11.8	7.6
PRO	3.9	5.3	6.8	5.2
Total	157.0	175.1	152.2	95.0

The genes encoding the synthesis of branched-chain amino acids were down-regulated, whereas four genes *lpdA, bkdA1A2*, SXYL_01340 organized in a cluster and involved in the catabolism of branched-chain amino acids were up-regulated (Table 1, Figure 1). The two genes (*bkdA1A2*) encode the subunits of branched-chain alpha-keto acid dehydrogenase E1 involved in the production of 3-methyl butanoyl-CoA, the precursor of 3-methyl butanoic acid (Table 1). The concentrations of leucine, isoleucine and valine decreased in the meat model after 48 h of incubation (Table 3). The pathway leading to the synthesis of 3-methyl butanoic acid, 3-methyl butanal and 3-methyl butanol from the catabolism of leucine has been characterized in *S. xylosus* (Beck et al., 2004). These aroma compounds and more generally methyl aldehydes, methyl acids and methyl alcohols contribute to the flavor of fermented sausages (Berdagué et al., 1993; Stahnke, 1995; Søndergaard and Stahnke, 2002).

Three genes involved in the aspartate metabolism were modulated in *S. xylosus* grown in the meat model, with two genes, *panD* and SXYL_01558, down-regulated, while *asnA* encoding L-asparaginase was up-regulated (Table 1). The gene *asnA* coding L-asparaginase in *L. sakei* was induced during the growth of this bacterium in a meat model and the corresponding mutant Δ*asnA2* showed reduced growth in this model, suggesting that asparagine could be a source of nitrogen (Hüfner et al., 2007).

Glutamate and glutamine were found in high concentrations in the meat model (Table 3). In contrast to the concentration of glutamate, which remained stable throughout incubation, the concentration of glutamine decreased sharply in the meat model (Table 3). Glutamate could be imported by the glutamate symporter protein encoded by *gltT* and catabolized by the glutamate dehydrogenases encoded by *gluD1* and *gluD2* (Table 1, Figure 1). Glutamate dehydrogenase activity provides α-ketoglutarate, which is required for amino acid transamination, which initiates the conversion of amino acids to aromatic compounds. Glutamate could be synthesized by the glutamate synthase from α-ketoglutarate, an intermediate of TCA cycle, and glutamine. This enzyme is encoded by the *gltBCD* cluster, which was overexpressed at 24 h of incubation (Table 1, Figure 1). Glutamate serves as the major amino group donor for all nitrogen-containing compounds, as a link between nitrogen and carbon metabolism. The glutamate synthesizing and degrading reactions must be tightly controlled to maintain its homeostasis (Gunka and Commichau, 2012). A cluster of four genes (SXYL_00105-108) was highly overexpressed at the three times of incubation (Table 1). This cluster is uncharacterized, but one of these genes potentially encodes a glutamine synthetase (*glnA2*). In *S. xylosus* C2a, another gene encodes a glutamine synthetase, *glnA1* located in a cluster with *glnR*. In *B. subtilis*, the *glnRA* operon is expressed in the absence of glutamine (Schreier et al., 1989). In the meat model, *glnR* and *glnA1* were not differentially expressed (Supplementary Table 2). Similarly, two genes encoding glutamine synthetases were identified in *Halobacillus halophilus*. In this bacterium, the expression of *glnA2*, but not *glnA1*, was increased in the presence of NaCl (Saum et al., 2006). The up-regulation of *glnA2* and the other genes of the cluster in *S. xylosus* could be linked to the osmotic stress generated by the presence of salt in the meat model.

### Response to osmotic stress generated by added NaCl

**Figure 2:**
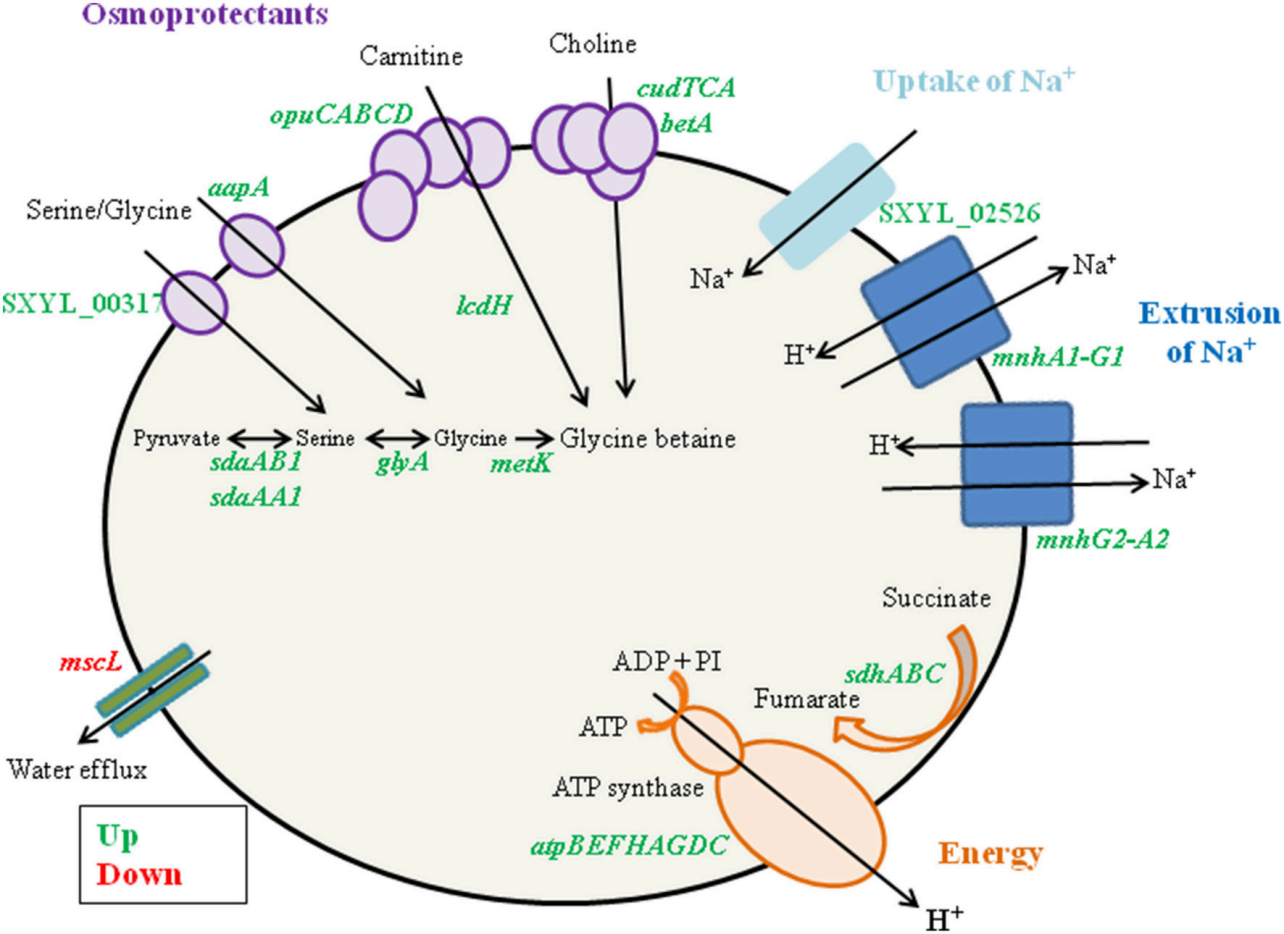
**Summary of the ***Staphylococcus xylosus*** response to osmotic stress generated by NaCl in a meat model**.

*S. xylosus* has to adapt to the high concentration of sodium chloride (0.47 M) in the meat model compared with 0.017 M in the medium used for the preparation of the inoculum. A primary response of *S. xylosus* to the presence of NaCl was the down-regulation of *mscL*, encoding a large conductance mechanosensitive channel, to prevent water efflux and maintain the physical integrity of the bacterial cells (Table 1, Figure 2).

In addition, genes encoding enzymes involved in three different pathways for the synthesis of glycine betaine, a powerful osmoprotectant, were up-regulated (Table 1, Figure 2). Among them, the cluster *opuC* (*opuCABCD*) and *lcdH* encoding L-carnitine dehydrogenase were identified. The cluster *opuC* encodes a glycine betaine/carnitine/choline ABC transporter identified as the only uptake route for L-carnitine in *B. subtilis* and *Listeria monocytogenes* (Kappes and Bremer, 1998; Angelidis and Smith, 2003). L-carnitine is found in raw meats with levels varying from 6.5 to 87.7 mg/100 g (Demarquoy et al., 2004). In the meat model, L-carnitine could be imported via the OpuC and catabolized to glycine betaine by L-carnitine dehydrogenase. The cluster of four genes (*cudTCA, betA*) encoding a choline transporter (*cudT*) and enzymes for dehydrogenation (*cudA, betA*) to form glycine betaine from choline were also up-regulated. These genes were transcriptionally regulated when *S. xylosus* C2a was cultured *in vitro* at high NaCl concentration (Rosenstein et al., 1999). Moreover, the genes *aapA* and SXYL_00317, which encode D-serine D-alanine glycine transporters, three genes (*sdaAA1, sdaAB1, glyA*) encoding enzymes involved in the synthesis of glycine, and *metK*, which encodes S-adenosyl-methionine, which serves as methyl donor for synthesis of glycine betaine, were up-regulated (Table 1, Figure 2). Concomitantly, we observed a sharp decrease of the concentrations of glycine and serine during the incubation in the meat model (Table 3).

The presence of NaCl induced the gene SXYL_02526 encoding a symporter of Na^+^ and the two clusters *mnh* (*mnhA1-G1, mnhG2-A2)* encoding Na^+^/H^+^ antiporter systems (Table 1, Figure 2). The modulation of *mnh2* expression was previously noted in *S. xylosus* grown in a meat model supplemented with nitroso compounds, the presence of nitrate and nitrite leading to the down-regulation of *mnh2* (Vermassen et al., 2014). In *S. aureus*, an active antiporter Mnh resulted in increased transcription of genes encoding respiratory chain components, such as the succinate dehydrogenase locus (*sdh*) (Swartz et al., 2007). The same observation was made in our meat model (Table 1). Moreover, all the genes involved in the synthesis of the multisubunit F_0_F_1_-ATPase were overexpressed by *S. xylosus*, as already mentioned (Table 1, Figure 2). This ATPase links the production of ATP to the transmembrane proton motive force (PMF) and can either generate ATP at the expense of the PMF or generate a PMF using ATP produced by fermentative substrate level phosphorylation (Cotter and Hill, 2003). The PMF can expel the protons, resulting from the activity of the two *S. xylosus* Mnh antiporters, from the cytoplasm to maintain pH homeostasis. In *Bacillus pseudofirmus*, proton pumping by the F_0_F_1_-ATPase generated a proton motive force across the membrane that powered Mnh antiporter activity after addition of Na^+^ (Morino et al., 2014).

Sigma factors play a role in the response of bacteria to environmental stress conditions. The expression of *rsbU* was up-regulated while *rsbV* and *rsbW* of the sigma B cluster were down-regulated (Table 1). In *S. epidermidis*, osmolarity increased the synthesis of polysaccharide intercellular adhesion (PIA) and biofilm formation. The induction of PIA synthesis in the presence of NaCl depends on a functional *rsbU* gene (Knobloch et al., 2001). Our results with the up-regulation of only *rbsU* in *S. xylosus* in the presence of salt could suggest a regulation independent of sigma B.

### Response to other stresses

*S. xylosus* overexpressed the cluster *dlt* involved in D-alanylation of lipoteichoic and wall teichoic acids (Table 1). The degree of D-alanylation varies depending on environmental conditions such as modification of pH or salt concentration (Neuhaus and Baddiley, 2003). Esterification in *S. aureus* was shown to increase with a decrease of pH (Neuhaus and Baddiley, 2003). Inactivation of *dltC* in *Streptococcus mutans* resulted in the generation of an acid-sensitive strain that could not grow below pH 6.5 (Cotter and Hill, 2003). In the meat model, *S. xylosus* has to adapt to acidification as the inoculum was grown at pH 7.0 before its inoculation in the meat model at pH 5.9 with a slight decrease during the incubation (Table 2). Thus, the up-regulation of *dlt* could be the result of adaptation to the acidic environment of meat. It is noteworthy that in the conditions of the meat model, the high concentration of NaCl (0.47 M) did not repress the expression of *dlt*, as reported for *S. aureus in vitro* conditions in the presence of 0.325 M NaCl (Koprivnjak et al., 2006).

Growth in the meat model did not seem to generate oxidative stress, as the genes *katC, katB, bsaA* and SXYL_00374 encoding, respectively, catalases, glutathione peroxidase and thioredoxin involved in the response to this stress, were down-regulated (Table 1). Whereas in our former study, nitrate and nitrate added to the meat model generated nitrosative stress that induced the up-regulation of genes involved in antioxidant defenses (Vermassen et al., 2014).

*S. xylosus* overexpressed the *crtPQMN* genes involved in the carotenoid biosynthesis pathway (Table 1). Moreover, eight genes (*mvaS, mvaCA, mvaK1DK2, fni, ispA*) encoding proteins of mevalonate pathway involved in the synthesis of farnesyl diphosphate, a precursor of the carotenoid synthesis, were up-regulated (Table 1). The C2a strain produces a yellow pigment after cultivation on agar medium, as do about 50% of *S. xylosus* strains (our unpublished data). *S. aureus* produced the intermediary yellow pigment 4,4′ diaponeurosporene, which is then converted to the yellow-orange end-product, staphyloxanthin, after prolonged cultivation (Wieland et al., 1994). In *S. aureus*, the CrtM and CrtN enzymes are responsible for the synthesis of the yellow pigment from farnesyl diphosphate (Wieland et al., 1994). Then, CrtP, CrtQ, CrtO, and AldH catalyze the oxidation, glycosylation and esterification reactions to convert this pigment into staphyloxanthin (Pelz et al., 2005; Kim and Lee, 2012). Staphyloxanthin protects *S. aureus* against oxidative stress by scavenging free radicals (Clauditz et al., 2006). But more generally carotenoids play a role in overall fitness (Clauditz et al., 2006; Johler et al., 2010) and could contribute to the growth and survival of *S. xylosus* in meat.

Finally, three genes encoding proteins that are enhanced when the cell is exposed to stress agents were down-regulated (Table 1). All these data suggest that the conditions of growth in meat did not seem to generate stress other than osmotic and acid for *S. xylosus*.

## Conclusion

The global gene expression of *S. xylosus in situ* in salted meat has allowed us to unravel its adaptation to this complex matrix in conditions that mimic the fermentation period of sausage manufacture. In these conditions, *S. xylosus* reached a plateau phase after 24 h and a balance between cell division and cell lysis was highlighted during this phase. *S. xylosus* adapted its metabolism to the meat nutrients and anaerobic conditions. It simultaneously used glucose and lactate as carbon sources. It used peptides and amino acids furnished by the meat. It has to cope essentially with the osmotic stress generated by addition of NaCl. It counteracted this stress by multiple strategies and particularly by the synthesis of glycine betaine, a powerful osmoprotectant.

## Author contributions

AV: Contribution to acquisition, analysis and interpretation of the data, contribution in drafting the article. ED: Contribution to experimental and data analysis, critical revising of the manuscript. AD: Statistical data analysis. PM: Contribution to data analysis. VL: Chemical analysis. SL, RT: equally contribute to this paper, design of the work, analysis and interpretation of data, writing of the manuscript

### Conflict of interest statement

The authors declare that the research was conducted in the absence of any commercial or financial relationships that could be construed as a potential conflict of interest.
