# Supplementary material for: Adaptation of Staphylococcus xylosus to Nutrients and Osmotic Stress in a Salted Meat Model
Source: Front Microbiol. 2016 Feb 5;7:87. doi: 10.3389/fmicb.2016.00087 (PMC4742526; doi:10.3389/fmicb.2016.00087)
Supplement: Supplementary file 1 [file Table1.DOCX]

**Supplementary Table 1. Targeted genes of *Staphylococcus xylosus* for the validation of microarray data by qPCR: expression at t24h, t48h or t72h in meat compared with inoculum.**

| **Locus tag** | **Gene** | **Sequences of primers pairs for qPCR (5'-3')** | | **Ratio of expression** | | | |  |  |
| --- | --- | --- | --- | --- | --- | --- | --- | --- | --- |
|  |  |  |  | **Microarray** | | | **qPCR** | | |
|  |  |  |  | **24 h** | **48 h** | **72 h** | **24 h** | **48 h** | **72 h** |
| SXYL_02533 | *katC* | TTCGATCATGAACGTATACCA | GTGTCTGGTGAACCTTTAGAG | 0.16 | 0.21 | 0.20 | 0.06 | 0.10 | 0.05 |
| SXYL_00749 | *sfaB* | GTCATTGATACATGCAACGCTA | TCGTCAGATGTTAATGGAAGCT | 0.14 | 0.17 | 0.20 | 0.04 | 0.04 | 0.03 |
| SXYL_02139 | *queC* | GGCTATCGTAGTGTTTAGTGGT | ATGTTGTTCTTCTGCAATGCG | 10.93 | 5.92 | 3.89 | 10.43 | 3.99 | 3.65 |
| SXYL_01803 | *pheT* | TAACGTGCTTGCTGAACGTAT | TGGCTCTACTTCACCAATGTC | 5.39 | 4.43 | 2.71 | 4.22 | 2.54 | 1.57 |
| SXYL_00869 | *leuC* | GCGACACTTGACCATAATGTG | CTGTTTCAGGTCCAACCATGT | 0.34 | 0.05 | 0.09 | 0.26 | 0.03 | 0.11 |
| SXYL_01084 | *metK* | GTTGCTTGTGAGACGACAGTA | GCCATTGTCTGGCTATCGTA | 6.76 | 8.33 | 4.81 | 7.66 | 13.09 | 9.31 |
| SXYL_02381 | *rplJ* | GTGGTCTAAGCGTTGCTGA | GTAGCAACTGCAGTAGGACC | 8.75 | 4.47 | 4.17 | 6.53 | 3.45 | 3.12 |
| SXYL_01987 | *dltD* | GGTACTGGTGGTTCGACAG | AACTGATTGAGCTGTGCCT | 8.13 | 6.11 | 3.22 | 14.45 | 9.53 | 4.32 |
| SXYL_01355 | *proC* | GTGCTGGAAATATGGCACATG | ATTGACGCCTAACTCATCAGC | 0.33 | 0.26 | 0.30 | 0.27 | 0.18 | 0.26 |
| SXYL_01317 | *gcvT* | GATGGGCTATGCCAGTCC | ACTGTGCAGCTTCACTACC | 0.27 | 0.29 | 0.50 | 0.16 | 0.18 | 0.30 |
| SXYL_02022 | *aroD* | TCGAACGAAGACTCAAGGTG | TTGCGATGCGCTGATTCTA | 0.25 | 0.27 | 0.33 | 0.07 | 0.08 | 0.10 |
| SXYL_00239 | *argC* | AGGTAGTGGTTATGGAGCGAT | GCGTTAAGTGACTCAAGTGAG | 0.42 | 0.27 | 0.37 | 0.24 | 0.09 | 0.09 |
| SXYL_00614 | *hutG* | CTGGCACAACGTGATCTTTG | TCAGGACCTTCTTTAGCACC | 0.08 | 0.24 | 0.33 | 0.03 | 0.17 | 0.12 |
| SXYL_02147 | *nagA* | GGACGCTTCATTTGAAGGATT | TCCCAACAATACTAGCTGCAT | 2.45 | 2.85 | 2.13 | 2.01 | 2.37 | 2.05 |
| SXYL_02148 | *fruA* | AGGAGGCTTTCCAGGACAA | GTGGACCAGGCGCATTAAT | 3.09 | 2.89 | 2.16 | 2.49 | 1.98 | 1.82 |
| SXYL_02415 | *folB* | TTATCATGGCGCGTTACCA | GGCTTCCCTTCCATAATCGC | 8.01 | 8.10 | 3.94 | 4.92 | 4.84 | 2.82 |
| SXYL_01655 | *fabD* | ATAGCTTAGGCGAGTATGCG | AACTTCCTCGTAATCCAAGCC | 10.51 | 8.43 | 4.99 | 11.45 | 11.35 | 5.05 |
| SXYL_01002 | *panD* | CATAGAGCGAGAGTGACGG | ACCACTACCTCGTTCACCTT | 0.37 | 0.19 | 0.25 | 0.24 | 0.06 | 0.07 |
| SXYL_00276 | *ldhB* | GGAAGAGTAGGTAGTCAAGTTTTAAC | CTAATAATGTACGATCGCCATCATT | 0.21 | 0.27 | 0.36 | 0.18 | 0.21 | 0.33 |
| SXYL_01576 | *glpD* | GCACAAGGTACAAGTTCACG | AAGCATACGTTCAGGTGTTGT | 0.14 | 0.16 | 0.20 | 0.08 | 0.09 | 0.10 |
| SXYL_00055 |  | TGATGGACACACACTTTATACTAAGA | ACAAGTTTGGATCATCCTTAGAGTC | 0.28 | 0.24 | 0.27 | 0.09 | 0.09 | 0.05 |
| SXYL_00270 | *ulaA* | GGTAACACAATCACTCGAACCATT | AAATGCTGCCATATAAAAGGTATGATG | 3.60 | 12.11 | 7.44 | 3.24 | 16.63 | 16.51 |
| SXYL_00784 | *czrA* | GGAAGATTCATTTAACGAACAAACG | TTGGTGAGAAACGTTAGATTGACT | 0.27 | 0.05 | 0.04 | 0.14 | 0.01 | 0.01 |
| SXYL_01098 | *ribE* | TGTTTACAGGAATTGTTGAAGAAATAGG | CTCTTTCTAAATTCACTTCAGCAGAT | 0.34 | 0.30 | 0.40 | 0.15 | 0.17 | 0.13 |
| SXYL_01961 | *argG* | TGGTTTAGATACAAGTGTCGCA | TGCATATGACACGAAATCTTCAC | 0.41 | 0.24 | 0.24 | 0.25 | 0.06 | 0.06 |
| SXYL_01974 | *mnhE1* | CGTCACAGGCAGTTATACATTTAATAA | CGGCTCATTATCAATCTTTGGTTT | 4.75 | 5.70 | 2.91 | 6.23 | 6.19 | 3.90 |
| SXYL_02467 | *sle1* | GACTCATTGTGGTCAATAGCGA | TGAAGACGTGTTCGAACTTGA | 5.68 | 5.22 | 6.09 | 4.97 | 2.78 | 6.64 |
| SXYL_00464 | *hisB* | CGTAACACTGCTGAAACACAATT | GTGACGTGGTGATCGTCTAC | 0.03 | 0.02 | 0.03 | 0.02 | 0.01 | 0.01 |
| SXYL_00466 | *hisD* | GATCCAGCGCTTAAAGCATATAAT | TTGATAAGTTTCAATACGAGCATGAC | 0.06 | 0.05 | 0.06 | 0.03 | 0.01 | 0.02 |
| SXYL_00467 | *hisG* | GCGTTAGAACAGAGAGAACGT | GCCAAATGGTAAGTCCAGAAG | 0.09 | 0.08 | 0.09 | 0.03 | 0.02 | 0.02 |
| SXYL_01501 | *trpD* | CATTCTATGTATGATCAACAGCCTT | GTAAATCTGTACTTCCTGATGATGAC | 0.45 | 0.40 | 0.46 | 0.16 | 0.18 | 0.11 |
| SXYL_00107 |  | GGTAAGCGAATTACTGGAGACTT | CCGTCTACGGAGTAAACATCA | 41.68 | 29.19 | 15.72 | 143.61 | 107.51 | 57.58 |
| SXYL_00108 | *gabD* | ATGGTGAGCAAATTCAAACTGAAA | AAGCATTAGCAGCGCTATCAA | 41.29 | 29.52 | 15.40 | 75.80 | 45.22 | 38.22 |
| SXYL_00173 |  | GGAAGTTGGATAGAGCCACAA | TTCTACACGATCCGCAATATCTAAT | 0.04 | 0.06 | 0.10 | 0.01 | 0.03 | 0.04 |
| SXYL_02642 |  | GAAGTAATATTCGATGCACATAGAGG | ACCTTCCAGTTTAGCTAGCTTT | 0.12 | 0.09 | 0.14 | 0.02 | 0.01 | 0.02 |
| SXYL_02644 | *metE* | TTACACAAAGAGAACTTATTACTCCAAA | ATATAGTGATAGTTTGTGTTGAACCATT | 0.12 | 0.13 | 0.19 | 0.02 | 0.01 | 0.02 |
| SXYL_02645 |  | ATTAGAACAATTAAAATCATCAACGTGG | AGTACCATACTGACTTACAATATTCCAA | 0.20 | 0.16 | 0.24 | 0.05 | 0.04 | 0.04 |
| SXYL_00255 |  | GACGTATGAAAATGGATAAGAATTTGATG | TCATCAGCAAAATAAGGAAATGCTTT | 0.03 | 0.08 | 0.18 | 0.00 | 0.05 | 0.13 |
| SXYL_00755 | *isdG* | GATGATGAGCAAGATTACGACC | CAGGTGACTCTTCACTGTTAGA | 0.16 | 0.21 | 0.30 | 0.23 | 0.21 | 0.41 |
| SXYL_01544 |  | AGTGTTAATGGTGACGATGATAC | TTGTTGTGAACTTTGTTCGCC | 2.30 | 5.22 | 5.92 | 2.39 | 8.02 | 17.92 |
| SXYL_00541 | *narJ* | GGAACGAGGGCAATTATTAGC | GGACTATTTTGCTCTGCCAATTG | 5.07 | 3.89 | 3.06 | 3.76 | 3.15 | 2.33 |
| SXYL_00540 | *narH* | GGCAGGTGGTCATGTTACT | CACGACAAGCCTCCTGATC | 6.50 | 4.62 | 3.73 | 4.34 | 4.08 | 2.15 |
| SXYL_01690 | *pyrP* | CACACCTTCGTTTCACTTGG | TGATACGCCATCACCGATG | 16.15 | 6.30 | 4.14 | 25.23 | 6.83 | 17.27 |
| SXYL_01934 | *spxA* | TCCGTATACGGAGCGTAACA | ACGACGTAATAAACCAGGGTT | 0.06 | 0.07 | 0.10 | 0.23 | 0.14 | 0.35 |
| SXYL_01041 | *hemG* | AGAACGTTTGGGTGATGAGG | CTGGGTATAATTGACGCTGC | 4.42 | 5.23 | 5.60 | 3.46 | 3.27 | 5.17 |
