# Supplementary material for: Adaptation of Staphylococcus xylosus to Nutrients and Osmotic Stress in a Salted Meat Model
Source: Front Microbiol. 2016 Feb 5;7:87. doi: 10.3389/fmicb.2016.00087 (PMC4742526; doi:10.3389/fmicb.2016.00087)
Supplement: Supplementary file 3 [file DataSheet1.DOCX]

**Supplementary Figure 1.** Comparison of log2 expression ratios of 47 differentially regulated genes measured by using microarray and qPCR at t_24h_ (A), t_48h_ (B) or t_72h_ (C). Positive and negative log2 expression ratios represent up- and down-regulation in a meat model *versus* the inoculum. Each data point is calculated from averages of biological triplicates.
